# Neural signatures of the response to emotional distraction: a review of evidence from brain imaging investigations

**DOI:** 10.3389/fnhum.2013.00200

**Published:** 2013-06-05

**Authors:** A. D. Iordan, S. Dolcos, F. Dolcos

**Affiliations:** ^1^Neuroscience Program, University of IllinoisUrbana-Champaign, IL, USA; ^2^Beckman Institute for Advanced Science and Technology, University of IllinoisUrbana-Champaign, IL, USA; ^3^Psychology Department, University of IllinoisUrbana-Champaign, IL, USA

**Keywords:** emotional interference, affective-cognitive interactions, amygdala, prefrontal cortex, working memory, neural circuitry, functional magnetic resonance imaging

## Abstract

Prompt responses to emotional, potentially threatening, stimuli are supported by neural mechanisms that allow for privileged access of emotional information to processing resources. The existence of these mechanisms can also make emotional stimuli potent distracters, particularly when task-irrelevant. The ability to deploy cognitive control in order to cope with emotional distraction is essential for adaptive behavior, while reduced control may lead to enhanced emotional distractibility, which is often a hallmark of affective disorders. Evidence suggests that increased susceptibility to emotional distraction is linked to changes in the processing of emotional information that affect both the basic response to and coping with emotional distraction, but the neural correlates of these phenomena are not clear. The present review discusses emerging evidence from brain imaging studies addressing these issues, and highlights the following three aspects. First, the response to emotional distraction is associated with opposing patterns of activity in a ventral “hot” affective system (*HotEmo*, showing *in*creased activity) and a dorsal “cold” executive system (*ColdEx*, showing *de*creased activity). Second, coping with emotional distraction involves *top–down* control in order to counteract the *bottom-up* influence of emotional distraction, and involves interactions between the amygdala and the prefrontal cortex. Third, both the response to and coping with emotional distraction are influenced by individual differences affecting emotional sensitivity and distractibility, which are linked to alterations of both *HotEmo* and *ColdEx* neural systems. Collectively, the available evidence identifies specific neural signatures of the response to emotional challenge, which are fundamental to understanding the mechanisms of emotion-cognition interactions in healthy functioning, and the changes linked to individual variation in emotional distractibility and susceptibility to affective disorders.

## Introduction

Emotion and cognition are two complexly intertwined, yet distinct facets of human behavior. Emotion has often been compared to a “double-edged sword,” as it can exert both beneficial and deleterious influences on our cognition and behavior. For example, we may experience enhanced memory for emotional events, but could also be more distracted by emotional stimuli that interfere with our goals. These effects have been linked to prioritization of emotional information, possibly due to its enhanced evolutionary value, as at a basic level these phenomena depend on neural mechanisms that allow timely detection, identification, and privileged processing of stimuli and situations that are important for survival (e.g., finding food, avoiding predators; Hansen and Hansen, 1988; Ledoux, 1996; Whalen et al., 1998b; Ohman et al., 2000, 2001; Anderson and Phelps, 2001).

Although the enhancing effects of emotion on cognitive functions such as memory, where emotion tends to be task-relevant, have been the focus of extensive research (see Dolcos et al., 2011, 2012 for comprehensive reviews), the detrimental effects of task-irrelevant emotion on cognitive functions have started to be the focus of research more recently (Johnson et al., 2005; Most et al., 2005; but see Seibert and Ellis, 1991; Oaksford et al., 1996; Shackman et al., 2006). An important factor modulating the impairing effect of emotion is the capacity to engage coping mechanisms in order to resist emotional distraction. Importantly, emotional distraction does not impact everybody in the same way, as people vary in their response to and the ability to cope with emotional distraction. This, in turn, influences the susceptibility to affective disorders, such as depression and anxiety, which are characterized by increased emotional distractibility. Thus, understanding the mechanisms underlying the response to and coping with emotional distraction is critical for understanding fundamentals of healthy functioning, as well as of changes associated with emotional disorders.

The present review discusses emerging evidence from brain imaging studies investigating the neural correlates of the detrimental impact of transient emotional distraction on goal-oriented processing and the neural correlates of coping with such distraction. The discussion focuses primarily on findings from studies using delayed-response working memory (WM) tasks and similar dual-task paradigms with emotional distraction, which allowed a clear dissociation of the fMRI signal in brain regions involved in cognitive and emotional processing. Although, overall, the focus in the present review is on the effect of transiently-induced emotional responses, in some cases investigations identified more complex combinations of effects, involving transient emotional responses, longer-lasting states, and trait-like aspects. For matters of conciseness, the present paper does not provide an in-depth discussion of evidence from studies employing perceptual, conflict resolution, and emotion regulation paradigms, which are also methodologically different (see Banich et al., 2009; Etkin et al., 2011; Shackman et al., 2011; Ochsner et al., 2012; Ray and Zald, 2012 for recent reviews and meta-analyses).

The focus will be on the following three main aspects: (1) We will first discuss evidence concerning the neural circuitry underlying the impact of emotional distraction, focusing on the interplay between two major neural systems: a ventral system associated with “hot” emotional processing (*HotEmo* system) and a dorsal system associated with “cold” executive processing (*ColdEx* system); (2) We will then discuss evidence concerning the neural mechanisms of coping with emotional distraction, focusing on the interaction between brain structures involved in basic emotional response (amygdala [AMY]) and brain structures involved in coping with irrelevant emotions (prefrontal [PFC] and anterior cingulate [ACC] cortices); (3) Finally, we will also discuss evidence concerning the role of individual differences in the response to and coping with emotional distraction in healthy participants, with a focus on personality and sex-related differences. The review will conclude with identification of outstanding issues emerging from the extant literature and discussion of future directions.

## Neural correlates of the response to emotional distraction—basic findings

### Neural correlates of the detrimental impact of emotional distraction

Investigations of the neural circuitry underlying the detrimental impact of emotional distraction complement the research investigating the neural correlates of the enhancing effect of emotion (reviewed in Dolcos et al., 2011, 2012). Studies investigating synergistic emotion-cognition interactions have revealed that the memory-enhancing effect of emotion is associated with *increased* activity in and interactions between emotion-based systems, involving AMY, and memory-based systems, involving medial-temporal lobe (MTL) and PFC regions (Dolcos et al., 2004; Kensinger and Corkin, 2004; see also Dolcos et al., 2011, 2012 for reviews). Based on the findings regarding the memory-enhancing effect of emotion, a default assumption concerning the impairing effect is that the detrimental impact of emotional distraction on cognitive functions may be linked to *reduced* activity in brain regions subserving the functions impaired by emotion. This assumption is supported by evidence from both clinical and non-clinical groups (Mayberg, 1997, 2006; Drevets and Raichle, 1998; Yamasaki et al., 2002; Price and Drevets, 2010, 2012).

**Figure 1:**
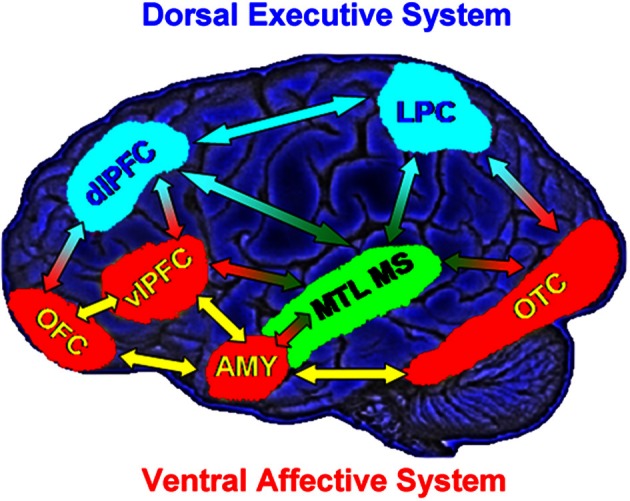
**Neural systems involved in cognitive/executive (dorsal) vs. emotional (ventral) processing**. The dorsal system includes brain regions typically associated with “cold” executive (*ColdEx*; color-coded in blue) functions, such as the dorsolateral prefrontal cortex (dlPFC) and the lateral parietal cortex (LPC), which are critical to the active maintenance of goal-relevant information in working memory (WM). The ventral system includes brain regions involved in “hot” emotional (*HotEmo*; color-coded in red) processing, such as the amygdala (AMY), the ventrolateral PFC (vlPFC), and the medial PFC. Other brain regions that these systems interact with (MTL MS, OTC) are also illustrated. MTL MS, medial temporal lobe memory system; OFC, orbitofrontal cortex; OTC, occipitotemporal cortex. Note that this diagram does not include all regions that are part of the two systems, as in its present format it does not include medial brain regions. Also, even though the visual cortical areas illustrated here (OTC) are not technically part of the *HotEmo* system, they are colored in red because they are susceptible to influences from emotion processing regions. Monochromatic arrows represent connections within the same system, whereas bichromatic arrows represent connections across systems. Adapted from figure courtesy of Dr. Lihong Wang and Dr. Aysenil Belger. Reproduced from Dolcos et al. (2011), with permission.

Models of affective-cognitive interactions inspired by clinical studies point to dysfunctional interactions between a dorsal executive neural system (*ColdEx*) and a ventral emotional system (*HotEmo*), and propose that impaired executive control and enhanced emotional distractibility observed in depression are linked to *hypo*function of the *ColdEx* and *hyper*function of the ventral *HotEmo* neural systems (Mayberg, 1997, 2006; Drevets and Raichle, 1998; Price and Drevets, 2010, 2012) (Figure 1). The dorsal *ColdEx* system includes brain regions typically associated with executive functions, such as the dorsolateral prefrontal cortex (dlPFC) and the lateral parietal cortex (LPC), which are critical to active maintenance of goal-relevant information in working memory (WM). Increased activity in these regions during WM tasks is typically associated with increased performance (Smith and Jonides, 1999; D'Esposito et al., 2000; Miller and Cohen, 2001; Nee et al., 2012; Niendam et al., 2012; Rottschy et al., 2012). The ventral *HotEmo* system includes brain regions involved in emotion processing, such as the AMY, the ventrolateral PFC (vlPFC), and the medial PFC (i.e., the medial aspect of the frontal lobe, excluding the ACC; Davidson and Irwin, 1999; Davis and Whalen, 2001; Phan et al., 2002; Kober et al., 2008; Vytal and Hamann, 2010; Lindquist et al., 2012).

**Figure 2:**
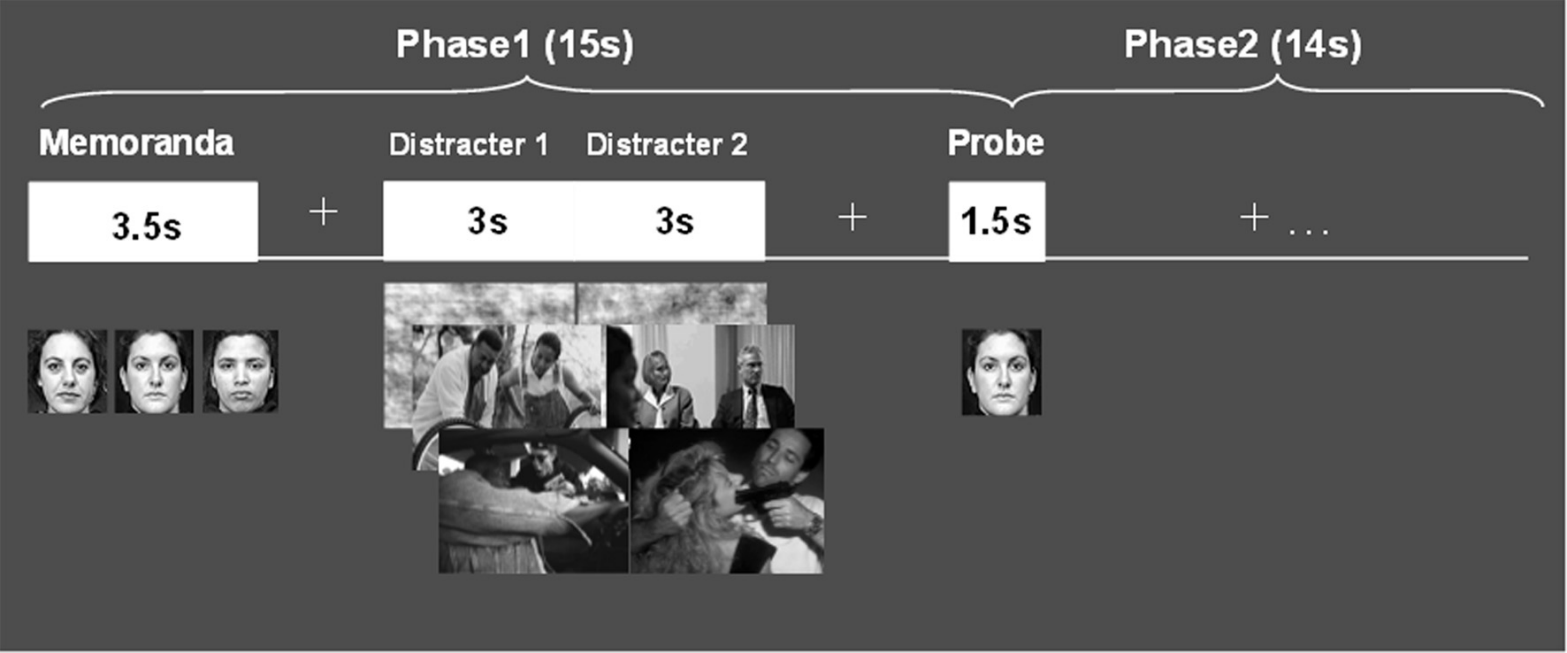
**Delayed-response WM task with emotional distraction**. The memoranda consisted of human faces, which participants encoded and maintained into WM. After a short delay, a probe was presented and subjects had to decide whether it was part of the memoranda or not. During the delay between the memoranda and the probes, meaningful (emotional and neutral) and meaningless (scrambled) novel pictures were presented on the screen, and subjects were instructed to maintain focus on the WM task while looking at the pictures. Reproduced from Dolcos and McCarthy (2006), with permission.

Findings from recent studies investigating the neural correlates of cognitive interference by emotional distraction in healthy participants provide evidence that interactions between the *ColdEx* and *HotEmo* systems are not only reflected in longer-lasting altered states, as observed in clinical conditions such as depression, but can also occur transiently, in response to on-going task irrelevant emotional distracters. A series of studies by Dolcos and colleagues, investigating the neural correlates of the response to emotional distraction, identified dissociable patterns of brain activity in *ColdEx* vs. *HotEmo* systems, which were specific to transient distracting emotions (Dolcos and McCarthy, 2006; Dolcos et al., 2007, 2008). The basic approach involved recording of brain activity using fMRI, while participants performed a delayed-response working memory (WM) task with emotional distraction (Figure 2; see also Wong et al., 2012 for a detailed presentation of the experimental protocol). The WM task involved keeping in mind a set of human faces (Memoranda) for the duration of a short delay, and then answering whether a single face (Probe) was part of the initial set or not. During the delay interval between the memoranda and the probe, high-arousing negative pictures, selected from the International Affective Picture System (IAPS; Lang et al., 2008), were presented as task-irrelevant distracters. The subjects were instructed to look at the distracters but maintain focus on the memoranda, and to make quick and accurate responses to the probes. Importantly, this task allowed clear dissociations of the time-course of response to emotional distraction in the *HotEmo* and *ColdEx* systems as well as an objective quantification of the impact of emotional distraction on WM performance.

**Figure 3:**
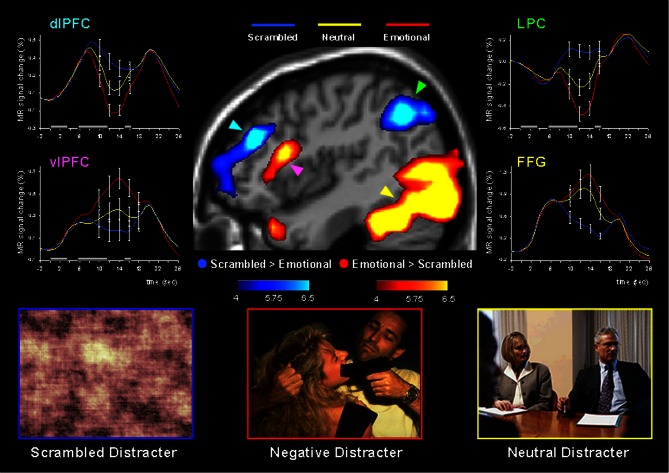
**Dissociable patterns of brain activity in the dorsal *ColdEx* and ventral *HotEmo* systems linked to impaired working memory performance in the presence of emotional distraction**. Emotional distracters produced the most disrupting effect on the activity during the delay period of a working memory task in a set of dorsal brain regions associated with executive processes (the blue clusters) while producing the most enhancing effect on activity in a set of ventral brain regions associated with emotion processing (the red clusters). The central image shows activation maps of the direct contrasts between the most versus least distracting conditions (i.e., emotional vs. scrambled pictures), superimposed on a high-resolution brain image displayed in a lateral view of the right hemisphere. The colored horizontal bars at the bottom of the brain image indicate the gradients of the t values for the activation maps displayed. The line graphs show the time courses of activity in representative dorsal and ventral brain regions (indicated by color-coded arrows). The gray rectangular boxes above the *x*-axes indicate the onset and duration of the different phases of the working memory task: presentation of the memoranda, distracters, and probes, respectively. PFC, Prefrontal Cortex; dlPFC, Dorsolateral PFC; LPC, Lateral Parietal Cortex; vlPFC, Ventrolateral PFC; FFG, Fusiform Gyrus. Reproduced from Dolcos and McCarthy (2006), with permission.

Using this paradigm, the study by Dolcos and McCarthy (2006) provided initial brain imaging evidence that impaired WM performance in the presence of emotional distraction is linked to increased activity in ventral system structures involved in emotional processing (e.g., AMY, vlPFC) while disrupting delay interval activity in dorsal brain regions implicated in attentional processes and active maintenance of task-relevant information in WM (e.g., dlPFC, LPC) (Figure 3). This opposing pattern of changes in *HotEmo* and *ColdEx* regions was confirmed by significant region × condition interactions (Dolcos and McCarthy, 2006). Importantly, the disruption of dorsal system activation was associated with impaired WM performance. The results of this study are consistent with the idea that activity in the affective and executive neural systems is interconnected, such that increased activity in the ventral affective regions in the presence of transient emotional distracters temporarily takes off-line the dorsal executive system and results in WM impairment, possibly as a result of a re-allocation of processing resources by emotional distraction (Dolcos and McCarthy, 2006).

**Figure 4:**
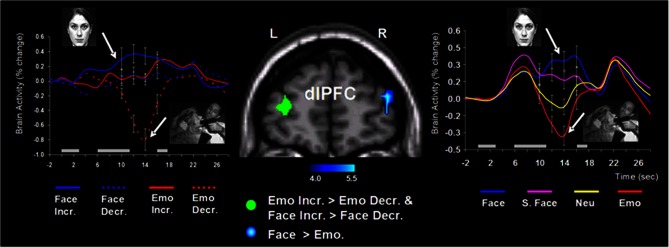
**Opposing dorsolateral prefrontal cortex (dlPFC) modulation linked to the nature of distraction**. Specific areas of the right dlPFC (e.g., BA 10/46) showed opposing modulation linked to the nature of distraction (i.e., *in*creased activity to memoranda-confusable face distracters, and *de*creased activity to emotional scene distracters). These findings were also confirmed when faces and emotional scene distracters were compared to their corresponding control conditions (i.e., scrambled faces and neutral scene distracters, respectively). The blue cluster on the middle panel shows the activation map of the direct contrast between delay activity to face and emotional distracters, superimposed on a high-resolution brain image displayed in a coronal view. The colored horizontal bar at the bottom of the brain image indicates the gradient of the t values. The line graph on the right side shows the time courses of activity in the right dlPFC region of interest (ROI). As described in section II below, specific dlPFC areas in the left hemisphere (i.e., the green cluster on the middle panel) showed similar modulation to face and emotional distraction linked to WM performance. The line graph on the left side shows the time courses of activity at peak voxels from overlapping areas of the left dlPFC (BAs 9/10) identified by analyses examining differences in brain activity associated with individual differences in performance in the presence of memoranda-confusable and memoranda-nonconfusable emotional distraction. For simplicity, the left-side graph is plotting the time courses of the face and emotional distracters alone (i.e., with the scrambled face and neutral conditions omitted). The gray rectangular boxes above the *x*-axes indicate the onset and duration of the memoranda, distracters, and the probes, respectively. Face, face distracters; S. Face, scrambled face distracters; Emo, emotional scene distracters; Neu, neutral scene distracters; Incr., increase group; Decr., decrease group; L, left; R, right; BA, Brodman area. In all graphs, error bars represent the standard errors of means. Reproduced from Dolcos et al. (2008), with permission.

Follow-up investigations (Dolcos et al., 2007, 2008; Denkova et al., 2010; Iordan et al., 2013b) provided additional evidence that these patterns of neural activity are specific to emotional distraction, and further explored the specificity of this response to different types of distracters. For instance, an investigation by Dolcos et al. (2008) directly compared the effects of novel non-emotional distracters that were highly similar to the memoranda (i.e., memoranda-confusable distracters) with those of emotional distracters, and showed that the two types of distracters were associated with opposing changes in dlPFC activity (i.e., increased vs. decreased, respectively), in conditions where both types of distracters produced similar effects on WM performance (see the activation cluster in the right hemisphere and the associated time course graph, in Figure 4 below). This provided support for the idea that dlPFC *de*activation is specific to emotional distraction (Dolcos et al., 2008).

Another recent study investigating the effects of more specific emotional distracters (i.e., anxiety-inducing angry faces), as opposed to those inducing a general emotional distraction involved in previous studies (i.e., IAPS pictures), found similar brain imaging effects (Denkova et al., 2010). Confirming that the manipulation worked in inducing anxiety, participants had significantly higher levels of state anxiety after the completion of the task compared to the beginning of the study. These findings show that similar dissociable patterns of activity in the *ColdEx* and *HotEmo* systems are also produced by relatively mild distracters (negative facial expressions) inducing specific emotions (anxiety; see also Grillon and Charney, 2011). Moreover, preliminary findings from an investigation that manipulated other emotional properties of task-irrelevant distracters (arousal: high vs. low, and valence: positive vs. negative) suggest that similar brain activity effects may also be observed in the case of positive distraction (Iordan et al., 2013b). Finally, other investigations using similar (Anticevic et al., 2010; Diaz et al., 2011; Oei et al., 2012) or different tasks (e.g., “emotional odd-ball task,” Yamasaki et al., 2002; Wang et al., 2005; “emotional interrupt task,” Mitchell et al., 2008), and evidence from clinical research (Morey et al., 2009; Anticevic et al., 2011) also support this dorso-ventral dissociation in response to emotional distraction, thus pointing to the replicability and generalizability of these findings (see Table 1 and Figure 10).

**Table 1:** **Relevant studies investigating the impact of emotional distraction, targeted in the present review**.

**Study**	**Subjects**	**Task**	**Memoranda**	**Type of emotional distracters**	**Behavioral results**	**Dorsal-ventral dissociation identified?**	**Additional areas**	**Coping with emotional distraction**	**Additional results**
Dolcos and McCarthy (2006)	F	WM with distraction	Faces	IAPS pictures	Decreased WM performance for Emo	Yes	Dorsal: PCC	Incr vlPFC activity associated with lower distractibility index	N/A
							Ventral: sgACC, Ins, HC		
Dolcos et al. (2006)^*^	F	WM with distraction	Faces	IAPS pictures	Decreased WM performance for Emo	Yes	N/A	Incr AMY-IFC/vlPFC coupling	N/A
								Incr L IFC/vlPFC for correct trials	
Dolcos et al. (2008)	F	WM with distraction	Faces	IAPS pictures	Subset of subjects showing decreased WM performance for Emo	Yes	Dorsal: SPC	Incr L dlPFC for subjects showing increased performance	Incr dlPFC for Face distracters
							Ventral: sgACC, MFC, HC		
Morey et al. (2009)	F + M, PTSD vs. CON	WM with distraction	Faces	Combat-related pictures	Decreased WM performance for PTSD group	Yes	Ventral: TP	N/A	Incr AMY, vlPFC, FG for Combat in PTSD group
									Decr dlPFC for Combat and Neu in PTSD group
Chuah et al. (2010)	F + M	WM with distraction	Faces	IAPS pictures	Decreased WM performance for Emo	Yes	Dorsal: dACC	Incr AMY-mPFC and AMY-dlPFC connectivity associated with better WM performance	Incr AMY associated with lower WM performance for Emo
					Decreased WM performance after sleep deprivation		Ventral: Ins		
Denkova et al. (2010)	F	WM with distraction	Faces	Morphed angry faces	Decreased WM performance for Emo	Yes^**^	Dorsal: dmPFC	Incr L vlPFC and dmPFC associated with increased WM performance	Incr L FG associated with increased social anxiety
							Ventral: vmPFC		Incr R FG associated with lower WM performance for Emo
Iordan et al. (2013a)	F + M	WM with distraction	Faces	Morphed angry faces	Decreased WM performance for Emo in F	Yes^**^	Dorsal: dmPFC	Incr L vlPFC associated with increased WM performance in F	Incr L FG to Emo in F, associated with lower WM performance
							Ventral: vmPFC		
								Incr R dlPFC associated with increased WM performance in M	Incr sgACC in F
Dolcos et al. (2013)	F	WM with distraction	Faces	IAPS pictures	Subset of subjects showing decreased WM performance to Emo	Yes	Dorsal: SPL	Incr L vlPFC for correct trials	Incr AMY and decr dlPFC in subjects showing impaired WM to Emo
							Ventral: mPFC, Ins, HC	Incr R vlPFC associated with WM success for subsequently remembered Emo distracters	Incr AMY associated with higher AI scores
								Incr dlPFC associated with lower AI scores
Anticevic et al. (2010)	F + M	WM with distraction	Shapes	IAPS pictures	Decreased WM performance for Emo	Yes	Dorsal: aPFC	Incr R aPFC and R vlPFC for correct trials	Decr aPFC and dlPFC associated with increased WM performance
							Ventral: OFC		
									Incr AMY associated with decreased WM performance for all distracter types
									Incr AMY-dlPFC negative coupling during task, compared to rest-state
Oei et al. (2012)	M	WM with distraction	Letters	IAPS pictures	Decreased WM performance and slower RT for Emo	Yes	Dorsal: aPFC	N/A	Incr ventral regions for stressed group
							Ventral: Ins, TP		
Yamasaki et al. (2002)^***^	F + M	Emotional odd-ball	N/A	IAPS pictures	Slower RT to Emo	Yes	N/A	N/A	Incr rACC to both Emo and targets
Wang et al. (2008b)^***^	F + M, MDD vs. CON	Emotional odd-ball	N/A	Commercial and in-house pictures	Slower RT to target-after-Emo	Yes	Dorsal: dACC, PCC	Incr ACC to target-after-Emo and incr R IFC to target-after-Neu in CON	Decr executive regions in MDD
					Slower RT for MDD, esp. to target-after-Emo		Ventral: Ins, sub-regional specificity in IFC	Incr R IFC associated with faster RT in MDD	

*The identification of the dorsal-ventral dissociation in the response to emotional distraction (column 7) was based on indexing brain regions typically showing decreased activity (dorsolateral prefrontal cortex [dlPFC] and lateral parietal cortex [LPC]) and brain regions typically showing increased activity (amygdala [AMY], ventrolateral prefrontal cortex [vlPFC], and fusiform gyrus [FG]) in response to emotional distraction. Of note, the ventral areas also included regions that are susceptible to emotion modulation (i.e., FG), that are not part of the emotional system *per se**.

* *Same subject sample as in the Dolcos and McCarthy (2006) study*.

** *There was no vlPFC activation identified in female participants, in this study*.

*** *Although these studies have not used the delayed WM task with distraction, they are based on a similar task (i.e., ‘emotional odd-ball’; introduced by the Yamasaki et al., 2002 study), which involves both executive and emotional processing components and dissociates between the dorsal and ventral networks (see also Wang et al., 2005, 2008a, 2012, for studies using similar tasks)*.

*Legend: F, female subjects; M, male subjects; PTSD, post-traumatic stress disorder clinical sample; MDD, major depressive disorder clinical sample; CON, healthy controls; WM, working memory, IAPS, International Affective Picture System (Lang et al., 2008); Emo, emotion; Neu, neutral; RT, reaction time; aPFC, anterior PFC; OFC, orbitofrontal cortex; IFC, inferior frontal cortex; mPFC, medial PFC; dmPFC, dorsomedial PFC; vmPFC, ventromedial PFC; MFC, medial frontal cortex; ACC, anterior cingulate cortex; rACC, rostral ACC; dACC, dorsal ACC; sgACC, subgenual ACC; PCC, posterior cingulate cortex, SPC, superior parietal cortex; SPL, superior parietal lobule; TP, temporal pole; Ins, insula; HC, hippocampus; Incr, increased activity; Decr, decreased activity; AI, attentional impulsiveness*.

Collectively, these findings are consistent with the idea that the outcome of task-irrelevant emotional distraction depends on dynamic interactions between neural systems that allow the ability to stay focused on task-relevant information and systems involved in the processing of emotional information that may compete with the available processing resources. Possibly as a result of their salience, emotional distracters may produce a *bottom-up* impact on processing of goal-relevant information by re-allocating processing resources (Vuilleumier et al., 2001) and impairing performance. Although the exact nature of these resources is not clear, one possible interpretation is along the lines of Desimone and Duncan's (1995) biased competition model of selective attention, consistent with the idea that processing of emotional stimuli requires attentional resources, and that emotional stimuli compete for neural representation with all the other stimuli. Hence, the emotional distracters tap into the same resources necessary to process the task-relevant information, and impair WM performance. It is possible, however, that processing of emotional, especially threatening, information is prioritized, and hence it occurs automatically, without being limited by the availability of attentional resources (e.g., Morris et al., 1999; Anderson et al., 2003). A potential reconciliation of these opposing views, in the perceptual domain, may be suggested by the results of a recent investigation from our group (Shafer et al., 2012), which showed that task-irrelevant emotion processing is subjective to both the emotional content of distraction and the level of attentional demand. Importantly, Shafer's et al. results showed that the interaction between emotion and cognition emerges only when finer assessments of emotional charge (comparison of most vs. least emotional conditions) along with manipulations of processing load (high vs. low) are taken into account, suggesting a more nuanced interplay between automatic and controlled processes involved in emotion processing (see also Van Dillen et al., 2009 and Vytal et al., 2012 for complementary approaches).

The opposing responses observed in the *HotEmo* and *ColdEx* systems in response to emotional distraction have proven to be robust and replicable results, demonstrated with different tasks, and also replicated by others. Similar bottom-up effects, consistent with the idea that emotional stimuli can “hijack” attention, have also been demonstrated using emotional variants of other cognitive tasks, tapping into perceptual and attentional domains (Williams et al., 1996; Bradley et al., 1999; Fox et al., 2001; Vuilleumier et al., 2001; Bradley, 2009; Cohen et al., 2011; Shafer et al., 2012). It should be noted that these studies have typically used emotional stimuli inducing transient emotions, such as emotional pictures and faces, and that these stimuli may have distinct characteristics compared to those typically employed in emotion-induction studies involving longer-lasting emotional responses (e.g., video clips and conditioned stimuli; see Okon-Singer et al., 2012 for a discussion). Moreover, as we will see in the next sections, further investigations also showed that this pattern of response to emotional distraction is sensitive to personality and sex-related differences (Denkova et al., 2010; Iordan et al., 2013a), affected by sleep deprivation (Chuah et al., 2010), and altered in clinical conditions, such as PTSD (Morey et al., 2009) and schizophrenia (Anticevic et al., 2011). Importantly, as described below, the disadvantageous outcomes of this bottom-up impact of emotional distraction can be mitigated by *top–down* interventions from cognitive control regions, engaged to regulate emotional responses and cope with emotional distraction (Gray et al., 2002; Dolcos et al., 2006, 2008; Pessoa, 2008; Chuah et al., 2010; Denkova et al., 2010; reviewed in Dolcos et al., 2011).

The dorsal-ventral dissociation in the neural response to emotional distraction has been observed not only in the larger neural systems (i.e., *ColdEx* and *HotEmo*), as discussed above, but also in more restricted brain areas, such as the ACC, which has been consistently associated with emotion-cognition integrations (Bush et al., 2000; Etkin et al., 2011; Shackman et al., 2011). A number of studies investigating conflict resolution by using emotional adaptations of cognitive conflict tasks (e.g., Stroop) point to a similar dorsal/ventral dissociation in the ACC, with the midcingulate cortex (“dorsal” ACC) responding mainly to cognitive conflict and perigenual-subgenual ACC (“rostral” ACC) responding mainly to emotional conflict (Whalen et al., 1998a; Etkin et al., 2006; Mohanty et al., 2007; also see Bush et al., 2000 for a review). However, other investigations have not fully supported this dissociation, offering a rather different picture, in which the dorsal ACC is engaged irrespective of the emotional content of the information to be ignored, whereas the ventral ACC remains selective for emotional information (Haas et al., 2006; Egner et al., 2008; Ochsner et al., 2009; Kanske and Kotz, 2011a,b). It should be noted that there are conceptual and methodological differences between studies employing delayed WM tasks with emotional distraction and studies involving cognitive-emotional conflict resolution (see Banich et al., 2009 for a discussion). Although it is beyond the scope of the present paper to discuss the latter type in detail, more in-depth discussions are provided in other recent reviews (Banich et al., 2009; Etkin et al., 2011; Shackman et al., 2011).

Noteworthy, the dorsal-ventral distinction is primarily a functional dissociation based on the opposing response to emotional distraction in identified typical cognitive/executive and emotion processing regions. In addition to this general dissociation, there are also exceptions, reflecting sub-regional specificity. For example, certain dorsal sub-regions show an increased response to emotional distraction (e.g., BA6/9; Dolcos et al., 2008). Also, as we will see in the next section, the increased response to emotional distraction in specific vlPFC areas has been linked to coping with emotional distraction (e.g., Dolcos et al., 2006). In other words, although consistent with its inclusion in the *HotEmo* system, vlPFC/inferior frontal cortex (IFC) shows overall increased activity to emotional distraction, consistent with evidence regarding its role in top–down control (Aron et al., 2004; Aron, 2007), specific areas within this larger region have proven to be involved in coping with emotional distraction. These results are consistent with other investigations that have implicated the dorsal PFC in emotion processing and the vlPFC in inhibition and affect regulation, respectively (see Aron, 2007; Kober et al., 2008; Vytal and Hamann, 2010; Ochsner et al., 2012 for recent reviews and meta-analyses).

In summary, studies investigating the neural correlates of the basic response to emotional distraction point to an interplay between two major neural systems: a ventral system, associated with “hot” emotional processing (*HotEmo* system), showing *in*creased activity, and a dorsal system, associated with “cold” executive processing (*ColdEx* system), showing *de*creased activity. The impact of task-irrelevant emotional distraction is chiefly supported by *bottom-up* mechanisms that may redirect processing resources away from the main cognitive task and toward stimuli with enhanced relevance for survival. As we will see in the next section, in response to this effect of task-irrelevant emotions, *top–down* mechanisms are engaged in order to cope with emotional distraction.

### Neural correlates of coping with emotional distraction

Brain imaging studies in which emotional information was presented as task-irrelevant distraction also provided evidence regarding the neural correlates of coping with distracting emotions. A series of investigations from our group and from others (Dolcos and McCarthy, 2006; Dolcos et al., 2006, 2008; Anticevic et al., 2010; Chuah et al., 2010; Denkova et al., 2010; Henckens et al., 2012; Oei et al., 2012) provided evidence that coping with task-irrelevant emotional distraction entails increased activity in and interactions between brain regions involved in basic emotion processing (AMY) and brain regions associated with cognitive control (particularly lateral and medial PFC). In this section we will discuss basic evidence concerning the role of the lateral PFC (mostly vlPFC) in coping with emotional distraction (see Table 1 and Figure 10), but the role of other regions (e.g., ACC) will also be emphasized. Complementary evidence concerning the neural correlates of coping with emotional distraction will be further elaborated in the section on individual differences. It is important to note that we operate a distinction between successful coping with emotional distraction and explicit manipulation of emotion regulation strategies, based on the different type of processing that is assessed in studies investigating the two aspects. Specifically, studies employing the delayed WM approach measure successful coping with emotional distraction *objectively*, in relation to performance in a cognitive task, whereas typical studies of explicit emotion regulation assess the effect of emotion regulation manipulation *subjectively*, in relation to emotional ratings. While here we discuss both objective and subjective aspects of coping with distraction, more in-depth discussions of the latter can be found in other sources (Gross, 2002; Gross and John, 2003; Ochsner et al., 2012; Ray and Zald, 2012).

#### Evidence of enhanced AMY-PFC coupling during processing of transient emotional distraction

**Figure 5:**
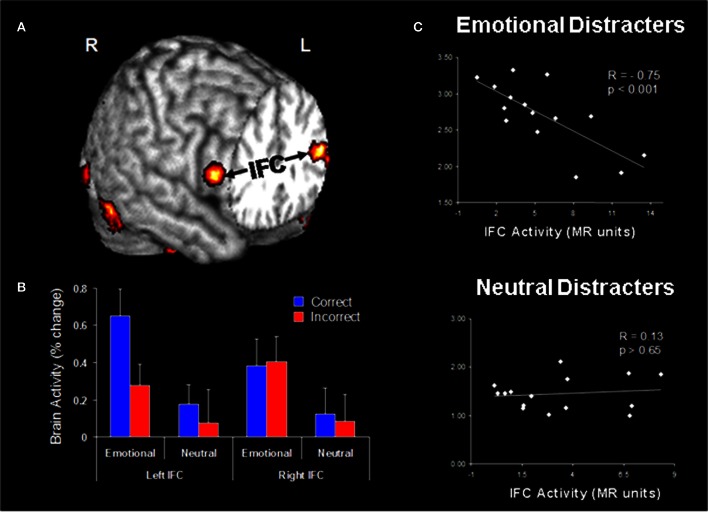
**Evidence for the role of lateral PFC in coping with distracting emotions. (A)** Brain regions showing enhanced functional coupling with the amygdala during processing of emotional distraction—ventrolateral prefrontal cortex (vlPFC)/inferior frontal cortex (IFC) highlighted. **(B)** Hemispheric asymmetry in the vlPFC/IFC during successful coping with emotional distraction. **(C)** Enhanced correlation between vlPFC activity and subjective emotional distractibility scores. Taken together, these findings suggest a hemispheric asymmetry in the IFC with respect to its role in actually coping with distraction (left vlPFC/IFC) vs. coping with the subjective feeling of being distracted (right vlPFC/IFC). Correct/Incorrect, Remembered/Forgotten items in the WM task; R, Right; L, Left. Adapted from Dolcos and McCarthy (2006) and Dolcos et al. (2006), with permission.

Functional connectivity analyses of data from the Dolcos and McCarthy study provided evidence for enhanced positive coupling between AMY and vlPFC/IFC during processing of emotional distraction (Figure 5A). In turn, the engagement of IFC leads to successful coping with emotional distraction, as reflected in greater activity to correct vs. incorrect trials in the WM task, despite the presence of emotional distraction (Dolcos et al., 2006). Further investigation of activity in these PFC regions provided evidence clarifying the consequences of their engagement in coping with emotional distraction (Figure 5B). The engagement of the AMY can be seen as having the role of an “emotional detector” that signals the PFC about the presence of emotional, potentially distracting, stimuli and thus the need to control their possible detrimental effects on cognitive performance (Dolcos et al., 2006). Anatomical evidence of substantial AMY–vlPFC connections (Amaral et al., 1992) supports this interpretation, and hence it is reasonable to posit that enhanced functional connectivity between the AMY and IFC reflects processing that originates in the AMY. Of all the lateral PFC regions, which are generally sparsely connected to the AMY, the IFC/vlPFC provides the most substantial connections, thus making it the best candidate among the lateral PFC regions to potentially exert direct control over AMY (Ray and Zald, 2012; see also Pessoa, 2010). Our interpretation is consistent with the idea that AMY is signaling the emotional relevance of the stimuli to PFC regions, such as ventrolateral and ventromedial PFC, which are integrating and interpreting them according to the current goals, and “taking” context-appropriate decisions which may dampen the emotional experience and benefit WM processing (Wager et al., 2008; Chuah et al., 2010; Denkova et al., under review). As described below, investigation of IFC activity in response to task-irrelevant emotional distraction provided further evidence consistent with this idea. These findings complement the results of emotion regulation studies typically identifying negative correlations between the levels of activity in PFC and AMY regions (e.g., Ochsner et al., 2004; see also Ray and Zald, 2012 for a review).

#### Evidence for the role of vlPFC/IFC in the inhibition of distracting emotions

Investigation of vlPFC/IFC activity in the two areas showing increased coupling with AMY in response to emotional distraction revealed a hemispheric asymmetry in this region, concerning its involvement in *objective* (left vlPFC) vs. *subjective* (right vlPFC) coping with emotional distraction. Specifically, activity in the left vlPFC distinguished between successful and unsuccessful WM trials in the presence of emotional distracters, by showing increased activity to trials associated with correct vs. incorrect responses. This finding suggests that this left vlPFC/IFC region is involved in successful coping with emotional distraction, by controlling the *objective* impact of emotional distraction on WM performance (Dolcos et al., 2006; Figure 5B). On the other hand, activity in the right vlPFC/IFC did not distinguish between correct and incorrect trials, but showed a negative correlation with subjective ratings of distractibility, for emotional but not for neutral distracters (Figure 5C). In other words, participants engaging this region during processing of emotional distracters perceived them as less distracting and less emotional, possibly as a result of engaging inhibitory processes that diminished the subjective experience of being distracted, thus pointing to a role of this region in coping with the *subjective* “feeling of being distracted” (Dolcos and McCarthy, 2006).

Overall, these findings are consistent with evidence pointing to vlPFC as a site of cross-modal inhibition, generally associated with inhibitory processes (Aron et al., 2004; Aron, 2007; Berkman et al., 2009) and with evidence associating vlPFC/IFC with the inhibition of negative emotions (Petrovic et al., 2002; Ochsner et al., 2004), in addition to ventromedial PFC (Diekhof et al., 2011). Also, as discussed in the section on the role of individual differences, subsequent investigations have further supported the role of the left PFC in coping with distracting stimuli conveying general (Dolcos et al., 2008) and specific (Denkova et al., 2010) negative emotions. Although the exact mechanism of interaction between these structures is not clear, a potential explanation for the dlPFC deactivation in response to emotional distraction could be based on AMY-driven bottom-up effects. By virtue of their salience, emotional distracters may trigger automatic reallocation of processing resources from the main cognitive task and impair WM performance (Anticevic et al., 2010; Chuah et al., 2010). Alternatively, it is possible that the actual mechanisms engaged in order to cope with emotional distraction (e.g., vlPFC-dependent) could trigger dlPFC deactivation, by tapping into a common regional pool of resources (Ray and Zald, 2012). This issue should be investigated in future studies.

Noteworthy, other investigations also point to the involvement of other brain regions, such as the ACC and dlPFC, in coping with emotional distraction. Regarding the ACC, the evidence consistent with the involvement of the ventral/rostral ACC in emotional conflict resolution also supports a role for this region in coping with irrelevant emotions (Bush et al., 2000; Etkin et al., 2006; Egner et al., 2008; Ochsner et al., 2009; Kanske and Kotz, 2011a,b). Regarding the dlPFC, other studies using adaptations of the Stroop task have rather emphasized the involvement of this region in coping with distraction (Compton et al., 2003; Herrington et al., 2005), consistent with a more generic role of the dlPFC in biasing processing toward task-relevant information and away from task-irrelevant information, irrespective of the emotional content of the information to be ignored (Banich et al., 2009).

In summary, the extant evidence concerning the neural correlates of coping with distracting emotion highlights the role of lateral PFC regions, particularly the left ventrolateral PFC, in diminishing the *objective* negative impact of irrelevant emotions on goal-oriented processing. The engagement of the ventrolateral PFC involves functional coupling with the AMY, which can be seen as an “emotional detector” signaling frontal regions about the need to control potentially distracting emotions. Other brain regions, such as the ventral ACC and the dlPFC, have also been linked to coping with emotional distraction, in the context of tasks requiring resolution of emotional conflict. As we will see in the next section, both the basic response to and coping with emotional distraction are influenced by individual differences, the investigation of which allows for a more refined understanding of the associated neural correlates.

## The role of individual differences in the response to emotional distraction

Investigation of individual differences is an important topic in the corpus of research examining emotion-cognition interactions (see Dolcos et al., 2011 for a review). Earlier investigations have linked various personality traits to differences in brain activity reflecting general and specific (e.g., anxiety) emotion processing (Canli et al., 2002b; Bishop et al., 2004; see also Hamann and Canli, 2004; Bishop, 2007 for reviews). Other studies identified sex differences in emotion processing, and pointed to specific differences in brain activity associated with enhanced emotional reactivity and emotional memory in women compared to men (Lang et al., 1993; Canli et al., 2002a; Cahill et al., 2004; see also Andreano and Cahill, 2009; Kret and De Gelder, 2012 for reviews). In the present section, we will review fMRI findings from studies investigating the role of individual differences linked to general aspects of cognitive/executive and affective domains (Dolcos et al., 2013), specific aspects of affective processing (i.e., anxiety; Denkova et al., 2010), and sex differences in both the basic response to and successful coping with transient emotional distraction (Iordan et al., 2013a). This line of investigation has been triggered by the Dolcos et al. (2008) study, which provided initial evidence for individual variation in the susceptibility to emotional distraction. Subsequent investigations further addressing this issue are discussed below. Investigation of these aspects is important for understanding emotion-cognition interactions in healthy functioning, as well as the changes linked to individual variation in emotional distractibility and susceptibility or resilience to affective disorders.

### Individual differences linked to general aspects of cognitive/executive and affective domains

The same study that identified the specificity of dlPFC engagement in response to emotional distraction (Dolcos et al., 2008) discussed above (see Figure 4) also provided evidence for the role of dlPFC in coping with distracting emotions. Results of this investigation revealed that, while in most participants emotional distraction impaired WM performance, in some subjects it did not have a detrimental effect. Analyses performed to examine the brain activity associated with individual differences in WM performance identified increased dlPFC activity in subjects whose performance was not impaired by the presence of emotional distraction (see Figure 4 above). However, given the lack of additional measures that could have further clarified the link between the observed behavioral and fMRI results in that study, it was difficult to assess what other factors may influence the differential sensitivity to emotional distraction. These issues were specifically targeted in a follow-up investigation (Dolcos et al., 2013), which in addition to fMRI data collected during the WM task with emotional distraction also assessed individual differences related to other aspects of general functioning in both cognitive/executive and affective domains, such as trait attentional impulsiveness and basic emotional sensitivity.

**Figure 6:**
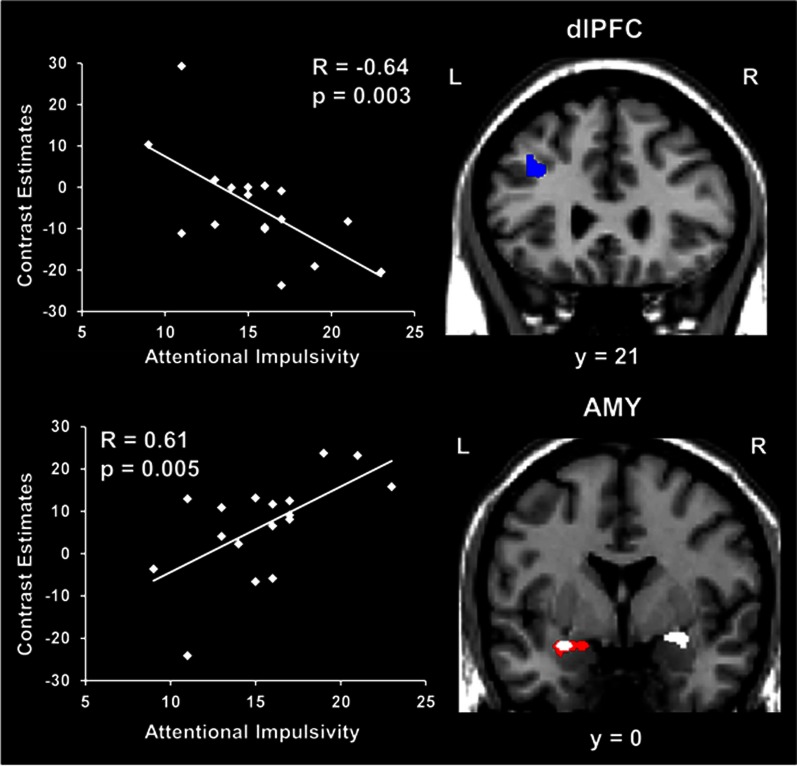
**Opposing co-variation of activity in *HotEmo* (AMY) and *ColdEx* (dlPFC) regions with individual differences in Attentional Impulsiveness**. Bilateral AMY activity increased (white clusters) and left dlPFC activity (BA 8/9) decreased (blue cluster) with individual scores of attentional impulsivity (AI). Interestingly, the positive correlation and the difference in activity overlapped in a left AMY area also showing increased activation in subjects showing impaired WM performance to emotional distraction (red cluster). Also, the positive co-variation identified at the group level in the right AMY was driven by the subjects showing impaired WM performance. The scatterplots illustrate the co-variation between brain activity in AMY and dlPFC to emotional vs. neutral distraction and AI scores. The activation maps are superimposed on a high resolution brain image displayed in coronal view (y indicates the Talairach coordinates on the anterior-posterior axis of the brain). AMY, Amygdala; dlPFC, Dorsolateral prefrontal cortex; L, Left; R, Right. Reproduced from Dolcos et al. (2013), with permission.

Regarding the basic response to emotional distraction, Dolcos et al. (2013) identified increased impact of irrelevant emotional distraction, affecting both *ColdEx* and *HotEmo* neural systems, in those subjects who showed increased susceptibility to emotional distraction. Specifically, participants who were more susceptible to the WM impairing effect of emotion showed greater *HotEmo* activations and greater *ColdEx* deactivations. For instance, the results identified increased AMY activation in subjects who were impaired by emotional distraction relative to those who were not (see Figure 6, the red cluster in the bottom panel depicting left AMY). These findings complement the results of the Dolcos et al. (2008) study, by showing that individual differences in the susceptibility to emotional distraction are associated not only with differences in top–down *ColdEx* regions (dlPFC), but also in ventral/bottom-up regions (AMY). Moreover, activity in both *HotEmo* and *ColdEx* regions was modulated by attentional impulsiveness. Specifically, trait attentional impulsiveness (AI), as assessed by the Barratt Impulsiveness Scale (Spinella, 2007), was associated with increased activity in the AMY and decreased activity in the dlPFC (Figure 6). Given the evidence that AI is characterized by increased distractibility and reduced ability to focus attention (Stanford et al., 2009), and the link between increased AI and impaired executive performance (Enticott et al., 2006; Pietrzak et al., 2008; Kam et al., 2012), this evidence points to AI as a potential general executive factor that contributes to increased sensitivity to emotional distraction.

Results from the same investigation also provided further support for the role of the left vlPFC/IFC in successful coping with emotional distraction, and revealed an interesting hemispheric dissociation between brain activity linked to the short-term vs. long-term impact of emotional distraction on WM and episodic memory (EM), respectively (Dolcos et al., 2013). Analyses of the fMRI data associated with WM success (by comparing the trials with correct vs. incorrect WM responses) identified increased left IFC activity, which provide further support for a role of this area in controlling the objective impact of emotional distraction (Dolcos et al., 2006). In contrast, analyses performed only on trials corresponding to distracters associated with WM success and which were later remembered during a subsequent EM task identified a similar pattern of increased response and a positive correlation with WM performance in the right vlPFC/IFC, suggesting a specific role of this area in linking the initial coping with emotional distraction with enhanced memory for the distracters themselves (Dolcos et al., 2013).

### Individual differences linked to specific aspects of affective processing: the role of anxiety

**Figure 7:**
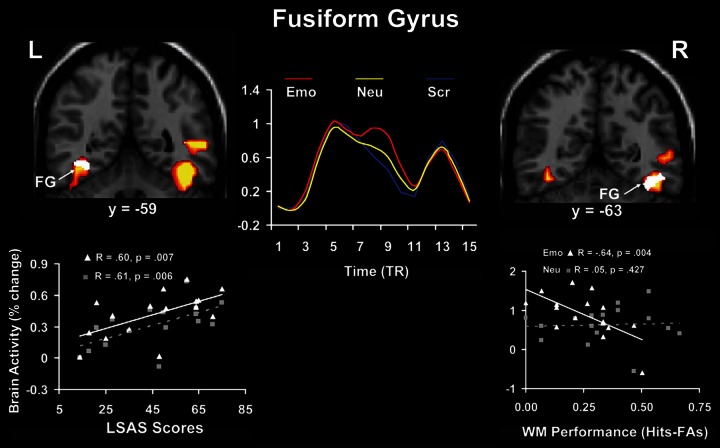
**Hemispheric asymmetry linked to bottom-up impact of emotional distraction in the fusiform gyrus (FG)**. Although these perceptual areas showed bilateral increased activity in response to anxiety-inducing distraction (red clusters and middle time course graph), a dissociation in the bottom-up response could be observed, linked to individual differences in trait anxiety and cognitive performance. Specifically, the left FG showed positive correlation with trait anxiety (white cluster in the left panel), whereas right FG showed negative correlation with working memory (WM) performance (white cluster in the right panel), consistent with a dissocation of subjective (left) and objective (right) effects. The middle panel illustrates the time course of activity in the FG, which was similar in both hemispheres. The scatterplots on the left and right panels are based on the corresponding correlations of the signal extracted from the FG with the social anxiety (LSAS) and WM scores, respectively. L, Left; R, Right; TR, Repetition Time. Adapted from Denkova et al. (2010), with permission.

Relationships between brain activity and personality-related differences were identified not only for traits reflecting general aspects of cognitive/executive and affective processing, but also for traits reflecting differences in processing and experiencing of specific emotions, such as anxiety. Complementing previous evidence showing that anxiety modulates the response to threat conveyed by social stimuli (e.g., angry faces) in primary emotion processing regions (AMY; e.g., Evans et al., 2008; Ewbank et al., 2009; see also Bishop et al., 2007), a recent study in healthy participants (Denkova et al., 2010) identified individual differences in brain activity linked to both the basic response to and coping with anxiety-inducing distraction (i.e., angry faces); for complementary approaches, see also Bishop (2009) and Ladouceur et al. (2009). Regarding the basic response to emotional distraction, the study by Denkova et al. (2010) identified a hemispheric asymmetry in the bottom-up impact of emotional distraction. Specifically, results pointed to a dissociation between the left and right fusiform gyrus (FG, BA 37), a perceptual region susceptible to emotion modulation (Kanwisher and Yovel, 2006), with the left FG showing positive correlation with anxiety scores and the right FG showing negative correlation with WM performance (Figure 7). This suggests a potential dissociation in the bottom-up impact of emotional distraction in the two hemispheres, with the left FG being involved in the subjective impact and experiencing of anxiety-inducing distraction and the right FG being involved in the actual/objective impact on WM performance.

In addition, medial prefrontal regions associated with experiencing of emotion (e.g., ventromedial PFC—vmPFC) showed increased overall activity and positive correlations with trait anxiety scores, whereas lateral regions associated with executive functions (e.g., dlPFC) showed decreased overall activity and negative correlations with trait anxiety scores (Denkova et al., 2010). Denkova et al. (2010) also found that activity in the same ventral and dorsal regions showing opposing changes to transient anxiety-inducing distraction (i.e., *in*creased vs. *de*creased activity) also showed opposing correlations with behavioral indices of trait anxiety and WM performance. Specifically, ventral regions showed patterns of positive correlation with trait anxiety and negative correlation with WM performance, whereas dorsal regions showed patterns of negative correlation with trait anxiety and positive correlation with WM performance. Although it is unclear how these regions interact with each other, these effects demonstrate that individual variations in trait anxiety and WM performance modulate the response to anxiety-inducing distraction in both ventral and dorsal regions. This complements previous evidence regarding the impact of task-irrelevant emotional distraction and points to more complex effects involving transiently-induced emotional responses and trait-like components, such as trait social anxiety. Finally, Denkova et al. (2010) also identified individual differences in coping with anxiety-inducing distraction. Consistent with the role of the left vlPFC in successful coping with task-irrelevant emotional distraction, results identified a positive correlation between activity in this region and WM performance, suggesting that participants showing less reduction in the left vlPFC activity (and hence, overall greater activity), also performed better in the WM task (see Figure 9 in the next subsection, left panel).

Overall, the results of these investigations suggest that individual differences in general cognitive/executive control (e.g., attentional impulsivity) and general and specific emotional sensitivity (e.g., anxiety) are linked to neural changes indexing increased sensitivity to emotional distraction, reflected in exacerbated activity in *HotEmo* regions and reduced activity in *ColdEx* regions, which affect both the basic response to and coping with distracting emotions. Given that previous investigations have revealed sex differences in the processing of emotional information (reviewed in Wager and Smith, 2003; Hamann and Canli, 2004; Stevens and Hamann, 2012), it was important to establish whether similar or different patterns of response would also be observed in women and men, in the context of delayed WM tasks with emotional distraction. A follow-up study using the same female subjects and methodology as in the Denkova et al. (2010) study, and adding a male sample, addressed these issues. The study by Iordan et al. (2013a) identified dissociable patterns of activity in the *HotEmo* and *ColdEx* networks in women and men, in the context of similar overall patterns of response to emotional distraction in the two sexes. Regarding commonly engaged areas, results showed that men and women display similar patterns of activation and deactivation in a host of brain regions associated with the ventral *HotEmo* (e.g., AMY, vmPFC, and FG) and dorsal *ColdEx* (e.g., dlPFC) neural systems, consistent with the idea of a generalizable pattern of response to emotional distraction across sexes. However, the study also identified differences in brain activity linked to differential impacts of and coping with emotional distraction in women and men. These results are featured in the next section.

### Sex differences in the response to emotional distraction

Available evidence has shown that in addition to enhanced emotional competence (Kring and Gordon, 1998; Seidlitz and Diener, 1998; Barrett et al., 2000), women also show enhanced reactivity to emotional challenge (Shields, 1991; Lang et al., 1993; Hamann and Canli, 2004), specificity in the deployment of emotion regulation strategies (Thayer et al., 2003; Matud, 2004; McRae et al., 2008; Mak et al., 2009; Domes et al., 2010; Denkova et al., 2012), and increased susceptibility to affective disorders (i.e., nearly two times higher lifetime prevalence of mood and anxiety disorders than men; Kessler, 2003; Bekker and Van Mens-Verhulst, 2007). Given the possibility that the same mechanisms that help generate the enhanced emotional experience in women could also be partially responsible for enhanced sensitivity to emotional factors, in a recent investigation (Iordan et al., 2013a) we examined whether sex-related differences in basic emotional reactivity are associated with differences in emotional distractibility, and identified neural mechanisms that implement differences in emotional distractibility between women and men.

**Figure 8:**
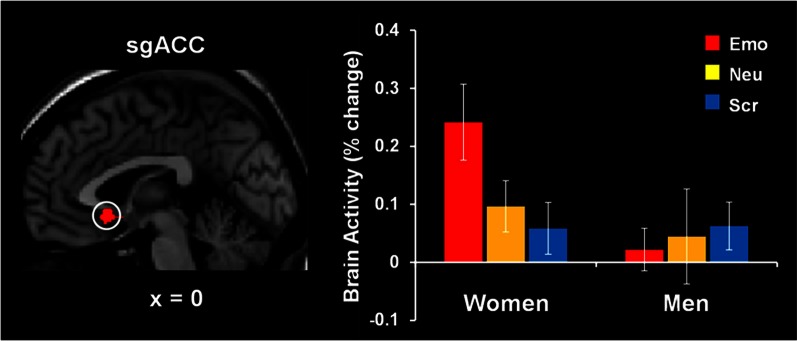
**Increased subgenual anterior cingulate cortex (sgACC) activity to emotional distraction, in women**. The area indicated by the white circle (BA 25), showing a difference in activation in response to angry faces in women versus men, was masked with a map identifying a main effect of emotion relative to baseline in women. The bar graph illustrates the fMRI signal, as extracted from the region of interest corresponding to the difference in activation between women and men. The activation map is superimposed on a high-resolution brain image displayed in sagittal view (with *x* indicating the Talairach coordinate on the left-right axis of the brain). Error bars represent standard errors of means. Emo, Emotional distracters; Neu, Neutral distracters; Scr, Scrambled distracters. Reproduced from Iordan et al. (2013a), with permission.

The study by Iordan et al. (2013a) identified sex differences in the basic response to emotional distraction, consistent with the idea of increased bottom-up impact of emotional distraction in women relative to men. Specifically, women showed increased sensitivity to emotional distraction in regions associated with the *HotEmo* system, such as FG, AMY, and subgenual ACC. Supporting the idea of enhanced bottom-up effects in female participants, the left FG, a perceptual area susceptible to modulation by emotion, showed a pattern of increased activity in response to angry-face distracters in women relative to men and negative correlation with WM performance in women only. These results complement the findings of our previous investigation in women (Denkova et al., 2010), in which a pattern of increased activity and negative correlation with WM performance was observed in the right FG (BA 37). Activity in the same right FG area, however, was not different and did not co-vary with WM performance in men. Given that FG is a region known to be selectively responsive to faces, the possibility that this effect might be more specific to emotional faces or to other emotional stimuli depicting human presence could not be excluded. An increased response to emotional distraction in women relative to men was also identified in the subgenual ACC (Figure 8), a higher-level emotion integration region, which has been linked to the experience of negative emotion, in both healthy and clinical samples (Gotlib et al., 2005; Mobbs et al., 2009; Baeken et al., 2010; Ball et al., 2012).

Interestingly, a specific pattern of sensitivity to emotional distraction was also revealed in men, who showed increased sensitivity in regions of the *ColdEx* system, including polar and dorsal PFC, and dorsal ACC, and in brain regions associated with the default mode network. However, overall WM performance was not affected by emotional distracters in the male participants, and overall they also had higher WM performance than the female subjects. Overall, these sex-related dissociations in the basic response to emotional distraction are consistent with increased sensitivity in “bottom-up” responses in women, linked to impaired WM performance, and increased sensitivity in “top–down” responses in men, linked to increased performance, in the face of emotional distraction. Noteworthy, these differences were identified in the context of overall similar response to emotional distraction in women and men, suggesting that, at a more general level, men and women also deploy similar mechanisms in response to transient emotional distraction.

**Figure 9:**
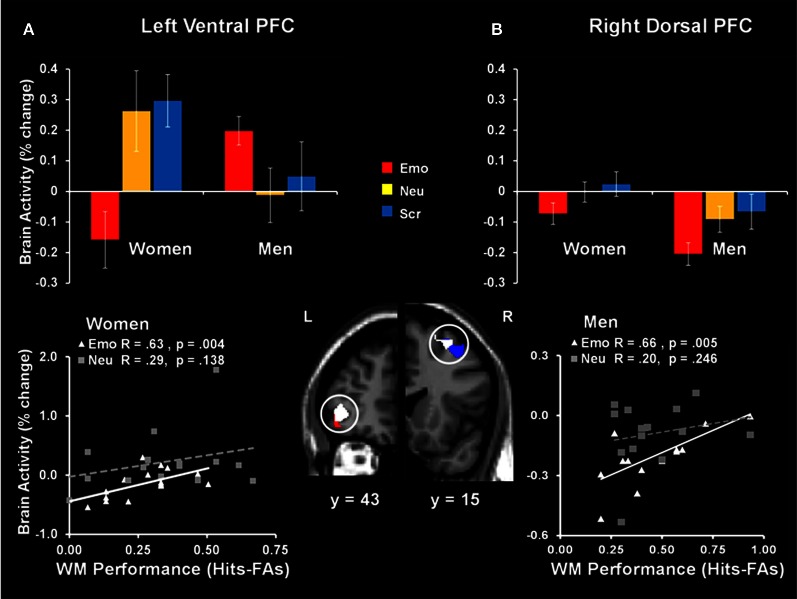
**Sex-related dorso-ventral dissociation in the lateral prefrontal cortex (PFC) in response to emotional distraction, linked to WM performance**. The left ventral PFC (BA 47) had overall reduced activity in women but showed increased activity in those women who coped successfully with emotional distraction **(A)**; a similar pattern was observed in the right dorsal PFC (BA 8/6) in men—although as a group they showed reduced activity in this region, compared to women, those who had increased activity also coped successfully with emotional distraction **(B)**. The bar graphs illustrate the fMRI signal, as extracted from regions of interest (ROI) corresponding to the differences in activation between women and men. The red and blue activation maps illustrate differences in the response to emotional distraction between women and men: men > women (red cluster) and women > men (blue cluster). The white activation maps illustrate the positive correlation between brain activity in response to emotional stimuli and WM performance in women (left ventral PFC) and men (right dorsal PFC). Scatterplots depicting these co-variations are presented in the bottom panels. The activation maps are superimposed on a high-resolution brain image displayed in coronal view (with y indicating the Talairach coordinates on the anterior- posterior axis of the brain). Error bars represent standard errors of means. Emo, Emotional distracters; Neu, Neutral distracters; Scr, Scrambled distracters; L, left; R, Right. Reproduced from Iordan et al. (2013a), with permission.

**Figure 10:**
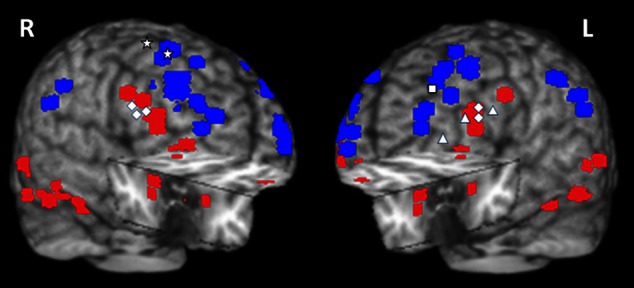
**Summary of activations in brain regions associated with the ventral *HotEmo* system (red) and the dorsal *ColdEx* system (blue)**. The figure shows peak activation voxels from areas showing increased (red) and decreased (blue) activity to emotional distraction, as identified by the studies featured in Table 1. The white geometric shapes identify peak voxels from regions associated with coping with emotional distraction, in women and men. Specifically, for the female subjects, the rhombi identify peak activation voxels from bilateral inferior frontal areas controlling for the subjective “feeling of being distracted” (Dolcos and McCarthy, 2006). The triangles identify peak activation voxels from left inferior frontal areas controlling for the objective impact of emotional distraction (Dolcos et al., 2006, 2013; Denkova et al., 2010); and the square identifies the peak activation voxel from a left dlPFC area linked to increased performance in the presence of emotional distraction (Dolcos et al., 2008). For the male subjects, the stars identify peak activation voxels in right dorsal frontal areas linked to increased performance in the presence of emotional distraction (Iordan et al., 2013a). The peak activation voxels are superimposed on a high resolution brain image displayed in a tridimensional view using MRIcro (www.mccauslandcenter.sc.edu/mricro/mricro/). R, Right; L, Left.

The same investigation also identified sex differences linked to the engagement of mechanisms to cope with emotional distraction. Results revealed a dorsal-ventral hemispheric dissociation within the lateral PFC, with the left ventral PFC being linked to individual differences in WM performance in women, and the right dorsal PFC being linked to individual differences in men (Figure 9). Interestingly, the same left vlPFC region showing enhanced activation in the female participants who performed better in the WM task (Denkova et al., 2010) showed “by default” an overall increased level of activity in males, who also had higher levels of WM performance. A similar pattern was observed in the right lateral PFC in men—although as a group they showed reduced activity in this region, compared to women, those who had increased activity also coped successfully with emotional distraction. The vlPFC results also bear relevance for the generalizability of the role of this region in coping with emotional distraction. Specifically, vlPFC's involvement in coping with emotional distraction has been supported mostly by results from studies with female participants (see Table 1 and Figure 10), and thus its role should be further clarified by future investigations comparing female and male participants. Overall, results of the two studies discussed above support the idea that enhanced emotional competence in women may have the side-effect of increased emotional reactivity, which in turn may lead to enhanced emotional distractibility. This phenomenon is reflected in different patterns of activity in response to emotional distraction in women relative to men, mainly consistent with an increased bottom-up effect of distracting emotions in women.

In summary, available evidence points to the role of individual differences in both the basic response to and coping with task-irrelevant emotions, suggesting that increased sensitivity to emotional distraction is associated with a pattern of activity characterized by both greater *HotEmo* activations and greater *ColdEx* reductions. Moreover, evidence also suggests that individual differences linked to general and specific aspects of cognitive/executive control and affective processing, such as trait attentional impulsiveness and anxiety, modulate the response to emotional distraction by increasing bottom-up *HotEmo* responses and diminishing top–down *ColdEx* engagement. Additionally, evidence points to sex differences in both bottom-up and top–down effects of emotional distraction, by linking increased recruitment of emotion processing areas with decreased cognitive performance in women and revealing dissociations in coping with distraction mechanisms between women and men. Finally, asymmetries between the left and right hemispheres linked to subjective vs. objective impact of emotional distraction on WM, resisting emotional distraction vs. coping and facilitation of long-term retention, and sex differences in coping with emotional distraction point to potential dissociations in their engagement in specific processes. Taking into account all these findings, it becomes clear that investigation of the role of individual differences that mediate the basic response to and the ability to cope with emotional challenge offers a promising path for better understanding of the susceptibility to affective disorders.

## Conclusions, open issues, and future directions

The present review discussed evidence regarding the neural correlates of the response to emotional distraction, as provided by fMRI studies focusing on three main topics: (1) the neural circuitry underlying the basic response linked to a detrimental impact of emotional distraction, (2) the neural mechanisms of coping with emotional distraction, and (3) the role of individual differences in these phenomena. Overall, the extant evidence points to specific neural signatures of the response to emotional challenge (summarized in Table 1 and Figure 10), which are fundamental to understanding the mechanisms underlying emotion-cognition interactions in healthy functioning, and the changes linked to individual variation in emotional distractibility and susceptibility or resilience to affective disorders. Regarding (1), the impact of task-irrelevant emotional distraction is associated with opposing patterns of activity in two major neural systems: a ventral system associated with “*hot*” emotional processing (*HotEmo system*), comprising regions such as AMY and vlPFC, which shows *in*creased activity, and a dorsal system associated with “*cold*” executive processing (*ColdEx system*), comprising regions such as dlPFC and LPC, which shows simultaneous *de*creased activity to emotional distraction. The reviewed evidence demonstrates that this is a robust pattern of activity, which has been systematically replicated using different types of tasks and stimuli. Moreover, this evidence suggests that the detrimental impact of task-irrelevant emotional distraction on goal-oriented processing is linked to *bottom-up* mechanisms, which are able to “hijack” processing resources and divert attention from the ongoing task to processing emotional information, which in turn leads to impaired cognitive performance.

Regarding (2), *top–down* control mechanisms are engaged in order to counteract the bottom-up influence produced by emotional distraction, cope with distracting emotions, and maintain cognitive performance. This interplay is supported by converging functional and anatomical evidence identifying specific roles for the involved structures, such as the AMY, acting as an “emotion detector,” and the PFC, particularly the vlPFC, acting as “top–down controller”; other regions, such as the ACC and medial PFC, are also involved. Noteworthy, recent evidence points to sex differences in the involvement of PFC in coping with emotional distraction, and further investigations are required to clarify whether the results based on female participants also generalize to males. Regarding (3), the behavioral responses linked to both the basic response to and coping with emotional distraction are influenced by individual differences, such as increased emotional sensitivity and distractibility, which are associated with greater *HotEmo* activations and greater *ColdEx* deactivations. Individual differences linked to both general and specific aspects of cognitive/executive and emotion processing, along with sex differences, also modulate activity in *HotEmo* and *ColdEx* systems. Overall, the findings regarding the role of individual differences point to a link between increased sensitivity to task-irrelevant emotional distraction and increased bottom-up effects. Finally, the reviewed evidence also points to hemispheric asymmetries seemingly linked to individual differences regarding specific processes, such as the experiencing of and coping with emotional distraction.

Despite a rapidly-growing body of literature providing clarification into the neural correlates of the response to emotional distraction, a number of issues are still unclear. Below, we briefly introduce them in relation to the topics covered in the present review.

1. An important open question refers to the role of emotional valence and arousal in the impact of emotional distraction. For instance, the majority of studies investigating the impact of task-irrelevant emotional distraction on performance in short-term/working memory tasks have used high-arousing negative distracters, and hence it is not known whether similar effects are also produced by positive distracters, or further dissociations linked to emotional arousal and valence exist. Given that positive and negative emotions have evolved to subserve different functions, it is reasonable to expect that their impact as distracters may be associated with different neural mechanisms, which may partially overlap with the mechanisms underlying the more general effect of emotional arousal. Only a limited number of studies have used stimuli with different emotional properties (i.e., arousal and valence) as task-irrelevant distracters, and the results so far have been mixed (Erk et al., 2007; Straube et al., 2008, 2011; Jasinska et al., 2012b). Preliminary findings from a recent investigation from our group (Iordan et al., 2013b) suggest that while “bottom-up” responses to emotional distraction engage mechanisms jointly sensitive to both arousal and valence (e.g., AMY), “top–down” responses are more specialized, with clearer dissociations between brain regions sensitive to arousal or valence.
2. Regarding the neural correlates of coping with emotional distraction, an important open question refers to understanding the role of different types of emotional control and their associated neural correlates. Although evidence from studies investigating the response to emotional distraction shows that the impact of task-irrelevant emotions is modulated by inhibitory mechanisms deployed in order to cope with distracting emotions (Dolcos and McCarthy, 2006; Dolcos et al., 2006), it has not been clear what type of coping with distraction strategies participants are using and whether there is a link between individual differences in coping with distraction and differences in emotion regulation strategies (Gross, 1998). Task manipulations emphasizing either the cognitive aspect of the task (consistent with an automatic engagement of coping mechanisms) or the engagement of more elaborate emotion regulation strategies (e.g., reappraisal) could potentially disentangle the outcomes of engaging automatic and controlled inhibitory processes on both emotional experience and cognitive performance.
3. Another open question refers to the role of individual differences in the impact of emotional distraction on different cognitive processes, other than WM. Although recent evidence reconciled a long-standing debate regarding whether the processing of emotional stimuli is automatic or depends on available attentional resources (Shafer et al., 2012; also see Lavie, 2005; Pessoa, 2005; Vuilleumier, 2005), by showing that task-irrelevant emotion processing is subjective to both the emotional content of distraction and the level of attentional demand, the role of individual differences in the impact of emotional distraction on lower-level perceptual processing has been less investigated (but see Bishop et al., 2004, 2007).
4. Investigations of the role of individual differences in the response to emotional distraction may prove informative not only for understanding features of individual variation in healthy subjects, but also changes associated with clinical conditions. Recent evidence suggests that dysfunctional alterations in factors influencing emotional sensitivity and susceptibility to emotional distraction, along with changes in the associated neural correlates, could play an important role in mental disorders affecting both emotional and cognitive domains, such as post-traumatic stress disorder (PTSD; Morey et al., 2009, 2011), depression (Wang et al., 2008a,b), and schizophrenia (Anticevic et al., 2011). For example, consistent with PTSD symptoms of hypervigilance and general distractibility during goal-directed cognitive processing, a recent investigation in PTSD patients (Morey et al., 2009) has identified increased activity in ventral processing regions related to trauma-related distracters and greater disruptions in cognitive processing regions. Also, combined behavioral-genetics (e.g., Jasinska et al., 2012a) and imaging-genetics investigations (Bishop et al., 2006; Morey et al., 2011; Qin et al., 2012) have highlighted the role of genetic differences in the response to emotional challenge. One such investigation in PTSD patients (Morey et al., 2011) identified increased responses to combat-related distracters in emotional processing regions, in the short allele carriers of the serotonin transporter gene. This evidence points to specific neural signatures of the response to emotional challenge, which may be used as neurobiological markers to enhance diagnostic accuracy and treatment efficacy.
5. Regarding the larger context of the impact of emotion on cognition, although a substantial corpus of research (reviewed in Dolcos et al., 2011) provides compelling evidence that emotion can produce both enhancing and impairing effects on cognition, the link between these two effects has been scarcely investigated (but see Shafer and Dolcos, 2012; Dolcos et al., 2013). Investigation of these effects within the same subjects is critical, as these opposing effects tend to co-occur not only in normal conditions but also in clinical disorders, such as PTSD and depression, characterized by alterations in both short- and long-term responses to emotional challenge. One of the few investigations of this issue (Dolcos et al., 2013) has examined the link between the short-term/impairing and long-term/enhancing effects of emotion in healthy subjects using a combined WM–EM paradigm, and identified dissociable bottom-up and top–down mechanisms of EM enhancement, linked to differences in the initial impact of emotional distraction on WM (i.e., WM impairment vs. successful coping with distraction). Further investigations of these phenomena should also include clinical samples (Dolcos, 2013).
6. Finally, manipulations involving other types of distraction, emerging from the engagement of other systems, such as long-term memory, could complement present evidence emphasizing the impact of transient visual distracters. This could also expand our present understanding of the role of individual differences in order to include a greater repertoire of responses and establish further links with changes occurring in clinical disorders. For example, clinical research has linked increased susceptibility to recollecting negative events with both symptom severity and cognitive impairment in emotional disorders such as depression and PTSD (e.g., Davis and Nolen-Hoeksema, 2000; Rubin et al., 2008). Hence, distressing thoughts related to personal events from the past and/or learned associations involving aversive stimuli could also act as powerful distracters even in healthy individuals, but it is not clear whether they engage the same neural systems as those associated with the response to visual emotional distraction.

### Conflict of interest statement

The authors declare that the research was conducted in the absence of any commercial or financial relationships that could be construed as a potential conflict of interest.
